# Long-Term Narrowband Lighting Influences Activity but Not Intrinsically Photosensitive Retinal Ganglion Cell-Driven Pupil Responses

**DOI:** 10.3389/fphys.2021.711525

**Published:** 2021-07-29

**Authors:** Linjiang Lou, Baskar Arumugam, Li-Fang Hung, Zhihui She, Krista M. Beach, Earl L. Smith, Lisa A. Ostrin

**Affiliations:** ^1^College of Optometry, University of Houston, Houston, TX, United States; ^2^Brien Holden Vision Institute, Sydney, NSW, Australia

**Keywords:** circadian rhythms, intrinsically photosensitive retinal ganglion cells, activity patterns, pupil, light exposure, rhesus monkey

## Abstract

**Purpose:** Light affects a variety of non-image forming processes, such as circadian rhythm entrainment and the pupillary light reflex, which are mediated by intrinsically photosensitive retinal ganglion cells (ipRGCs). The purpose of this study was to assess the effects of long- and short-wavelength ambient lighting on activity patterns and pupil responses in rhesus monkeys.

**Methods:** Infant rhesus monkeys were reared under either broadband “white” light (*n* = 14), long-wavelength “red” light (*n* = 20; 630 nm), or short-wavelength “blue” light (*n* = 21; 465 nm) on a 12-h light/dark cycle starting at 24.1 ± 2.6 days of age. Activity was measured for the first 4 months of the experimental period using a Fitbit activity tracking device and quantified as average step counts during the daytime (lights-on) and nighttime (lights-off) periods. Pupil responses to 1 s red (651 nm) and blue (456 nm) stimuli were measured after approximately 8 months. Pupil metrics included maximum constriction and the 6 s post-illumination pupil response (PIPR).

**Results:** Activity during the lights-on period increased with age during the first 10 weeks (*p* < 0.001 for all) and was not significantly different for monkeys reared in white, red, or blue light (*p* = 0.07). Activity during the 12-h lights-off period was significantly greater for monkeys reared in blue light compared to those in white light (*p* = 0.02), but not compared to those in red light (*p* = 0.08). However, blue light reared monkeys exhibited significantly lower activity compared to both white and red light reared monkeys during the first hour of the lights-off period (*p* = 0.01 for both) and greater activity during the final hour of the lights-off period (*p* < 0.001 for both). Maximum pupil constriction and the 6 s PIPR to 1 s red and blue stimuli were not significantly different between groups (*p* > 0.05 for all).

**Conclusion:** Findings suggest that long-term exposure to 12-h narrowband blue light results in greater disruption in nighttime behavioral patterns compared to narrowband red light. Normal pupil responses measured later in the rearing period suggest that ipRGCs adapt after long-term exposure to narrowband lighting.

## Introduction

Light plays a critical role in a variety of physiological, psychological, and behavioral functions that are important for mental and physical health (Emens and Burgess, 2015; Prayag et al., 2019). Light is required to synchronize the biological clock to the 24-h day (Prayag et al., 2019). Moreover, light directly impacts the sleep/wake cycle through suppression of melatonin release to increase alertness (Lewy et al., 1980; Cajochen, 2007; Emens and Burgess, 2015). The influence of light exposure on these non-image forming functions is dependent on the specific properties of the light, including the timing and duration of exposure, intensity, and spectral composition. Irregular light exposure patterns can lead to disturbances in sleep and circadian rhythm regulation (Duffy and Czeisler, 2009; Prayag et al., 2019). The non-image forming responses to light are primarily facilitated by intrinsically photosensitive retinal ganglion cells (ipRGCs; Berson et al., 2002; Hattar et al., 2002).

The intrinsic, light-sensitive characteristics of ipRGCs are mediated by the photopigment melanopsin, which has a peak sensitivity of approximately 482 nm (Berson et al., 2002; Dacey et al., 2005). Additionally, the ipRGCs receive extrinsic synaptic input from rod and cone photoreceptors (Dacey et al., 2005). The ipRGCs project to the suprachiasmatic nucleus, the brain area involved in circadian rhythm entrainment (Hattar et al., 2006; Hannibal et al., 2014). Light input received by the suprachiasmatic nucleus is ultimately relayed to the pineal gland to regulate melatonin release (Moore, 1996; Emens and Burgess, 2015). The ipRGCs also project to the olivary pretectal nucleus, which is involved in the pupillary light reflex (Hattar et al., 2006; Hannibal et al., 2014). The pupillary light reflex can be used to assess ipRGC activity *in vivo*. The initial, rapid pupil constriction observed after light onset is primarily controlled by rod and cone photoreceptors (Kardon et al., 2009), whereas the sustained pupil constriction observed after short-wavelength light offset, termed the post-illumination pupil response (PIPR), is primarily controlled by intrinsic melanopsin activity (Gamlin et al., 2007; Adhikari et al., 2015).

Given the spectral sensitivity of ipRGCs, short-wavelength light is more effective for suppressing melatonin secretion and phase shifting the melatonin rhythm compared to middle- or long-wavelength light (Brainard et al., 2001; Thapan et al., 2001; Lockley et al., 2003). In humans, exposure to short-wavelength light at night increases alertness, which has been associated with melatonin suppression (Cajochen et al., 2005; Figueiro et al., 2007). In contrast, reducing exposure to short-wavelength light in the evening for 2 weeks has been shown to increase objectively measured melatonin levels and sleep duration, as well as subjectively measured sleep quality in humans, which has been suggested to be due to decreased stimulation of the ipRGCs before bedtime (Ostrin et al., 2017). However, reducing daytime exposure to short-wavelength light for 4 weeks does not alter the sleep/wake rhythm or evening melatonin levels (Domagalik et al., 2020), suggesting that the circadian system can adapt to changes in the spectral composition of environmental light.

Long-wavelength light, which does not directly activate the ipRGCs, can also affect various physiological and behavioral responses. In humans, short-term exposure to long-wavelength light was shown to increase subjective and objective alertness and increase performance during the daytime, without inducing melatonin suppression (Figueiro et al., 2009; Sahin and Figueiro, 2013; Sahin et al., 2014). Moreover, in rodents, it was demonstrated that exposure to a long-wavelength light/dark cycle results in greater diurnal locomotor activity and reduced nocturnal activity compared to a short-wavelength light/dark cycle (van der Merwe et al., 2019). Studies investigating the effects of the wavelength of light on physiological, psychological, and behavioral responses have primarily focused on short-term exposures, or increasing or reducing exposure to light in the evening. Thus, the impact of long-term exposure to a short- or long-wavelength light/dark cycle on circadian activity has not been extensively studied.

The rhesus monkey is a good model for studying circadian rhythms because it is a diurnal species and displays activity and sleep patterns similar to that of humans (Daley et al., 2006; Hsieh et al., 2008; Masuda and Zhdanova, 2010). Rhesus monkeys exhibit a diurnal pattern of activity under a regular light/dark cycle, with high activity during the daytime (i.e., the lights-on period) when monkeys are awake and low activity during the nighttime (i.e., the lights-off period) when monkeys are expected to be sleeping (Weed et al., 1997; Masuda and Zhdanova, 2010). The onset and offset of activity in monkeys tend to occur close to light onset and offset, respectively (Masuda and Zhdanova, 2010). Therefore, activity rhythms of rhesus monkeys entrain to the light/dark cycle. However, how the spectral composition of ambient lighting affects this entrainment is unknown. The purpose of this study was to determine the effects of short- and long-wavelength ambient lighting on activity patterns and rod/cone- and ipRGC-mediated pupil responses in infant rhesus monkeys that were part of studies examining the effects of narrowband ambient lighting on eye growth (Hung et al., 2018, 2020).

## Materials and Methods

### Subjects and Lighting Conditions

Subjects were infant rhesus monkeys (*Macaca mulatta*), obtained at 2–3 weeks of age. Control monkeys (*n* = 14) were housed under broadband “white” fluorescent lighting (Philips TL735, CCT = 3,500 K; Philips Lighting, Sommerset, NJ, United States) with an average illuminance of 480 lux (range = 342–688 lux; for husbandry details, see Smith and Hung, 1999). The spectral composition of the white fluorescent lighting contained multiple peaks with maximum intensities at 550 and 612 nm, and smaller peaks at 430 and 490 nm (She et al., 2020). Experimental monkeys (*n* = 41) were initially housed under white fluorescent lighting. At 24.1 ± 2.6 days of age, the experimental monkeys were transferred to a separate room (3 m × 4.6 m) illuminated with either long-wavelength “red” light (*n* = 20; 630 nm; half-max bandwidth of 20 nm) or short-wavelength “blue” light (*n* = 21; 465 nm; half-max bandwidth of 20 nm). Red light rearing conditions have been described previously (Hung et al., 2018). Briefly, red and blue lighting were produced with ceiling mounted light emitting diodes (LEDs; Philips ColorGraze MX4 Powercore lighting system, Philips North America, Andover, MA, United States). The intensities of the red and blue LEDs were adjusted to produce equal irradiances. The red and blue lighting systems produced an average energy level of 1.39 W/m^2^, as measured with a CL 500A illuminance spectrophotometer (CL 500A; Konica Minolta Sensing America, Inc., Ramsey, NJ, United States) in the middle of the housing area at the height of the junction between the upper and lower cages. The average illuminances for the red and blue lighting systems, expressed in human lux, were 274 ± 64 and 183 ± 28 lux, respectively. The room contained multiple individual cages and a group socialization area. All monkeys were maintained on a 12-h light/12-h dark cycle, with the lights-on cycle beginning at 7:00 AM. Procedures were approved by the Institutional Animal Care and Use Committee at the University of Houston and conformed to the ARVO statement for the Use of Animals in Ophthalmic and Vision Research.

### Daily Physical Activity Measures

Eight monkeys reared under white fluorescent light, 12 monkeys reared under red light, and 15 monkeys reared under blue light were fitted with a Fitbit activity tracking device (Fitbit Flex; Fitbit, Inc., San Francisco, CA, United States) starting from 28.4 ± 10.7 days of age and monitored until 150.5 ± 5.9 days of age. Fitbits were mounted on lightweight helmets that were worn by the monkeys holding optical lenses that were part of a different study (Hung et al., 2018). The Fitbits were removed for charging every 3–4 days. For monkeys reared under blue light, data were collected for the entire experimental period. However, for most monkeys reared under white or red light, data were collected only during weeks 1–10 and 17–18. On average for all lighting conditions, valid Fitbit data were available for 59.2 ± 12.7 days during the data collection period from weeks 1–10 and 17–18. Monkeys reared under blue light wore the device more consistently throughout the experimental period compared to monkeys reared under white or red light. Step counts during the lights-on and lights-off periods were recorded as a measure of activity.

### Pupillometry

The pupillometry protocol has been described previously (Ostrin et al., 2018). Pupillometry testing was performed at 267.7 ± 50.4 days of age for eight white light reared monkeys, 20 red light reared monkeys, and 18 blue light reared monkeys. All pupillometry measurements were conducted in the morning to minimize potential effects of circadian variation (Zele et al., 2011; Münch et al., 2012). Monkeys were anesthetized with an intramuscular injection of 10 mg/kg ketamine and 1 mg/kg acepromazine, supplemented with a half dose approximately every 10 min. This relatively low dose of anesthesia was used to immobilize the monkeys, while minimizing sympathetic system suppression from anesthesia to allow the pupil to be fully responsive to light stimulation. Heart rate and blood oxygen were monitored with a pulse oximeter (model 9847V; Nonin Medical, Inc., Plymouth, MN, United States). Heart rate was maintained at approximately 180–200 beats/min, similar to the awake state, indicating that the sympathetic system was not significantly suppressed. The left eye was dilated with 1% tropicamide. Monkeys were placed in a head holder, and the eyelids were held open with a speculum. Custom made plano powered rigid gas permeable contact lenses were placed in each eye with moisturizing lubricant (Refresh Celluvisc, Allergan) to maintain corneal integrity and optimize quality for pupil imaging.

**Figure 1:**
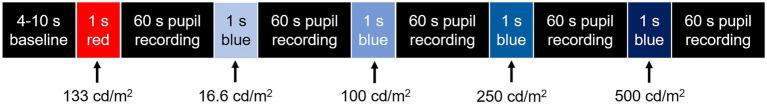
Pupillometry protocol. Baseline pupil diameter was recorded in the dark for 4–10 s. A 1 s red stimulus (133 cd/m^2^) and four increasing intensities of 1 s blue stimuli (16.6, 100, 250, and 500 cd/m^2^) were presented, with a 60 s interstimulus interval.

Stimuli were presented to the left eye with an LED-driven Ganzfeld system (Color Burst, Espion, Diagnosys LLC, MA, United States) positioned approximately 10 mm in front of the eye. The consensual pupil response was recorded in the right eye with an infrared eye tracker at 60 Hz (ViewPoint EyeTracker, Arrington, AZ, United States). The camera was focused at the pupil plane and calibrated before the start of each session. Baseline pupil diameter was recorded in the dark for 4–10 s prior to the onset of the first stimulus. A 1 s long-wavelength “red” stimulus (133 cd/m^2^, 3.3 × 10^14^ photons/cm^2^/s) was presented first, followed by four 1 s short-wavelength “blue” stimuli with increasing intensities, 16.6 cd/m^2^ (6.4 × 10^13^ photons/cm^2^/s), 100 cd/m^2^ (3.7 × 10^14^ photons/cm^2^/s), 250 cd/m^2^ (9.2 × 10^14^ photons/cm^2^/s), and 500 cd/m^2^ (1.5 × 10^15^ photons/cm^2^/s), with an interstimulus interval of 60 s (Figure 1). The red stimulus was 651 nm with a half-max width of 25 nm, and the blue stimuli were 456 nm with a half-max width of 20 nm (Spectroradiometer CS1W; Minolta, Tokyo, Japan).

### Data Analysis

Fitbits were synced every 7 days, and activity data (step counts) were downloaded from the Fitbit website. Step counts recorded at 15-min intervals were manually entered into Excel. Data for the red light and blue light reared monkeys were organized relative to the number of days from the start of the red or blue light rearing period. Data for the white light reared monkeys were age matched to the red light and blue light reared monkeys. For each monkey, total step counts during the 12-h lights-on and 12-h lights-off periods were calculated for each day. Average 12-h lights-on and light-off step counts were calculated and binned into 2-week intervals, starting from week 1 (i.e., the first week under red or blue light rearing) until week 18. Data were binned and averaged into 2-week intervals to allow us to examine changes in activity over time with fewer missing data points and to group age into categorical values. Weeks 11–16 were excluded from the analysis because there was no activity data collected during this time period for the majority of monkeys. Because lights-off activity significantly differed between groups, total step counts during the lights-off period were further analyzed in bins, including the first hour (7–8 PM), middle 10 h (8 PM–6 AM), and final hour (6–7 AM) of the lights-off period. The first hour, middle 10 h, and final hour of the lights-off period were analyzed separately to determine whether the observed differences in activity occurred throughout the entire lights-off period.

Pupil data were analyzed offline using a custom written MATLAB program. Samples deemed poor quality by the Viewpoint software were removed from the pupil trace. Baseline pupil diameter, maximum constriction, and the 6 s PIPR were calculated. Baseline pupil diameter was calculated as the average pupil diameter during the 4–10 s recording period prior to the onset of the first stimulus. Relative pupil sizes were calculated by dividing the pupil diameter by the baseline pupil diameter. Maximum constriction was calculated as the difference between the baseline pupil diameter and the minimum pupil diameter during light stimulation. The 6 s PIPR was calculated as the difference between the baseline pupil diameter and the average pupil diameter 6–7 s after stimulus offset. Both maximum constriction and the 6 s PIPR are presented as percent difference from baseline. Expressing pupil metrics relative to baseline reduces the dependence of the pupil metrics on baseline pupil diameter and minimizes the effects of inter-individual differences in baseline pupil diameter (Joyce et al., 2016; Kelbsch et al., 2019). Three control monkeys reared under white fluorescent light underwent repeat testing 2–3 months after their first measurement to assess repeatability of the pupil measures. Repeatability data were assessed with Bland-Altman analysis (Bland and Altman, 1986).

### Statistical Analysis

Statistical analyses were performed with IBM SPSS Statistics version 27 (IBM, Armonk, NY, United States). For activity data, lights-on and lights-off step counts were analyzed separately, and linear mixed model analysis with restricted maximum likelihood estimation was performed with fixed factors condition and week. Heterogeneous first-order autoregressive covariance structure was used for the repeated factor week. *Post hoc* tests were corrected for multiple comparisons using the Benjamini-Hochberg correction. For pupil response data, pupil metrics between white, red, and blue light reared monkeys were compared using a one-way ANOVA or Kruskal-Wallis test for non-parametric data. Normality was assessed with the Shapiro-Wilk test. Value of *p* < 0.05 was considered statistically significant.

## Results

**Figure 2:**
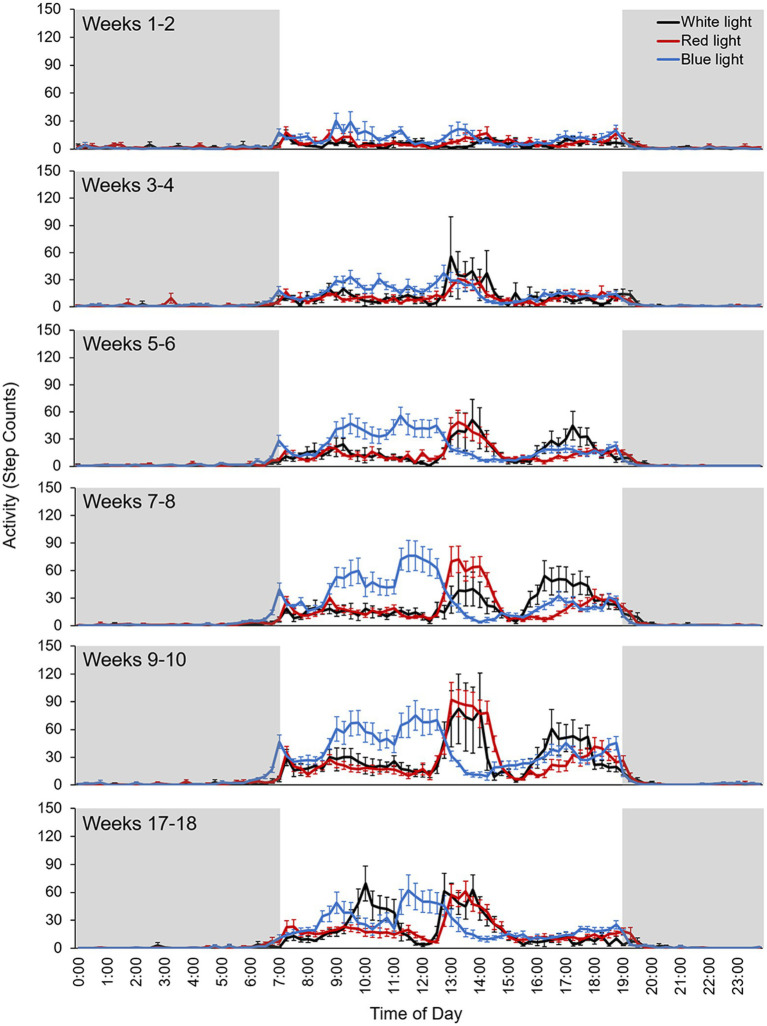
Averaged daily activity cycles for each 2-week bin for monkeys reared in white light (black line), red light (red line), and blue light (blue line). Step counts were recorded at 15-min intervals. Error bars represent SEM. Shaded areas indicate the lights-off period.

Activity data for the 12-h lights-on and lights-off periods for white, red, and blue light reared monkeys for each 2-week bin from weeks 1–10 and 17–18 are shown in Table 1. Averaged daily activity cycles for each 2-week bin for monkeys reared in white, red, and blue light are shown in Figure 2. Across the entire experimental period, activity counts during the lights-on period for monkeys reared in white, red, and blue light were 942 ± 185, 877 ± 166, and 1,215 ± 152, respectively, and activity counts during the lights-off period were 49 ± 12, 67 ± 10, and 91 ± 18, respectively.

**Table 1:** Average 12-h lights-on (7 AM–7 PM) and lights-off (7 PM–7 AM) activity (step counts) for white, red, and blue light reared monkeys by 2-week bins.

Week	Lights-on activity (step counts)			Lights-off activity (step counts)		
	White light (*n* = 8)	Red light (*n* = 12)	Blue light (*n* = 15)	White light (*n* = 8)	Red light (*n* = 12)	Blue light (*n* = 15)
Weeks 1–2	301 ± 32	376 ± 59	566 ± 119	86 ± 24	77 ± 14	63 ± 10
Weeks 3–4	620 ± 135	535 ± 79	798 ± 88	41 ± 8	63 ± 11	92 ± 27
Weeks 5–6	836 ± 109	734 ± 122	1,119 ± 123	35 ± 10	62 ± 6	91 ± 12
Weeks 7–8	1,121 ± 128	1,084 ± 163	1,510 ± 139	50 ± 11	74 ± 5	104 ± 14
Weeks 9–10	1,464 ± 231	1,401 ± 221	1,842 ± 174	46 ± 11	67 ± 12	132 ± 19
Weeks 17–18	1,055 ± 118	953 ± 105	1,192 ± 156	40 ± 6	57 ± 13	52 ± 11
Average (all weeks)	942 ± 185	877 ± 166	1,215 ± 152	49 ± 12	67 ± 10	91 ± 18

Data are represented as mean ± SEM.

### Lights-On Activity

**Figure 3:**
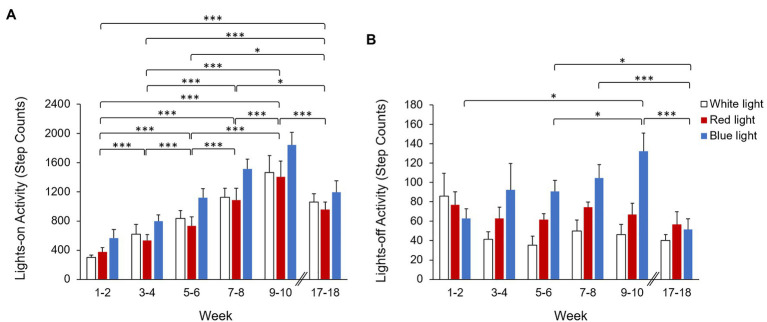
Average activity (step counts) during the **(A)** 12-h lights-on period (7 AM–7 PM) and **(B)** 12-h lights-off period (7 PM–7 AM) by 2-week bins for monkeys reared in white light (open bars), red light (red bars), and blue light (blue bars). Activity during the lights-on period increased with age from weeks 1–2 to 9–10, but was not significantly different between monkeys reared in white, red, or blue light. During the lights-off period, monkeys reared in blue light exhibited significantly greater activity compared to monkeys reared in white light. Error bars represent SEM. ^*^p < 0.05 and ^***^p < 0.001 for pairwise *post hoc* comparisons between weeks.

For activity during the 12-h lights-on period (7 AM–7 PM), linear mixed model analysis revealed a significant main effect of week (*p* < 0.001), but no significant main effect of condition (*p* = 0.07). The interaction between condition and week was not significant (*p* = 0.63; Table 2). During the 12-h lights-on period, activity increased with age during the first 10 weeks of the experimental period (*p* < 0.001 between all 2-week bins), with no differences in activity between monkeys reared in white, red, or blue light. By weeks 17–18, overall lights-on activity decreased compared to weeks 9–10 (*p* < 0.001). The lowest lights-on activity was observed during weeks 1–2, and the greatest lights-on activity was observed during weeks 9–10 (Figure 3A).

**Table 2:** Results from linear mixed model analysis of the activity data for the factors condition and week, and the interaction of condition by week.

	*p*		
	Condition	Week	Condition × Week
12-h lights-on activity (7 AM–7 PM)	0.07	<0.001^*^	0.63
12-h lights-off activity (7 PM–7 AM)	0.01^*^	0.003^*^	0.02^*^
First hour of the lights-off period (7–8 PM)	0.004^*^	0.49	0.63
Middle 10 h of the lights-off period (8 PM–6 AM)	0.22	0.001^*^	0.38
Final hour of the lights-off period (6–7 AM)	<0.001^*^	<0.001^*^	<0.001^*^

* *p* < 0.05.

The activity plots show that the maximum peak of activity in blue light reared monkeys occurred earlier in the day compared to white and red light reared monkeys (Figure 2). However, all monkeys had approximately 1.5 h of group playtime each day and the duration of this increase in activity corresponds to the duration of the group playtime. Thus, the differences in the timing of the activity peaks in blue light reared monkeys compared to white and red light reared monkeys likely reflect differences in the timing of the group playtime schedules. In addition, the activity plots show that the onset and offset of activity occurred close to the time of light onset and offset, respectively. However, the onset of activity in blue light reared monkeys occurred earlier, before light onset, compared to white and red light reared monkeys during the first 10 weeks of the experimental period.

### Lights-Off Activity

For the 12-h lights-off period (7 PM–7 AM), activity significantly differed based on condition (*p* = 0.01) and week (*p* = 0.003), and there was a significant interaction effect between condition and week (*p* = 0.02; Table 2). Overall, monkeys reared in blue light exhibited significantly greater lights-off activity compared to white light reared monkeys (*p* = 0.02). Twelve-hour lights-off activity did not significantly differ between blue light and red light reared monkeys (*p* = 0.08) or between red light and white light reared monkeys (*p* = 0.24; Table 1).

*Post hoc* pairwise comparisons on the interaction effect revealed that during weeks 1–2, 3–4, and 17–18, there were no significant differences in 12-h lights-off activity between white, red, and blue light reared monkeys (*p* > 0.05 for all). During weeks 5–6 and 7–8, blue light reared monkeys exhibited significantly greater activity compared to white light reared monkeys (*p* = 0.003 and *p* = 0.006, respectively), but not compared to red light reared monkeys (*p* = 0.06 and *p* = 0.07, respectively). Blue light reared monkeys also exhibited significantly greater activity during weeks 9–10 compared to both white light and red light reared monkeys (*p* = 0.009 and *p* = 0.01, respectively). There was no significant difference in 12-h lights-off activity between red light and white light reared monkeys for any 2-week bin (*p* > 0.05 for all). Twelve-hour lights-off activity for monkeys reared in blue light significantly differed based on week, with the greatest activity observed during weeks 9–10 and the lowest activity observed during weeks 17–18. For blue light reared monkeys, activity during weeks 9–10 was significantly greater than activity observed during weeks 1–2, 5–6, and 17–18 (*p* < 0.05 for all). Activity during weeks 17–18 was also significantly lower than activity exhibited during weeks 5–6 and 7–8 (*p* < 0.05 for both; Figure 3B). Monkeys reared in white light and red light did not exhibit significant changes in activity with week (*p* > 0.05 for all).

**Figure 4:**
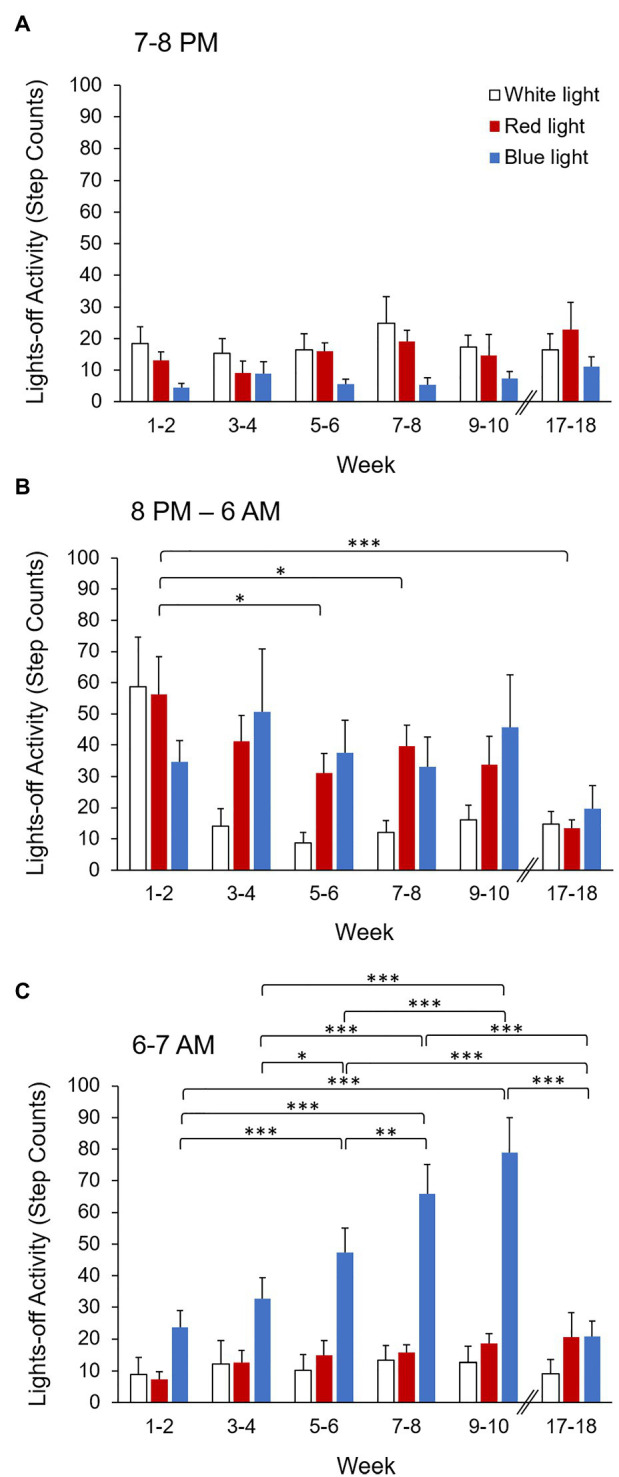
Activity (step counts) during the **(A)** first hour (7–8 PM), **(B)** middle 10 h (8 PM–6 AM), and **(C)** final hour (6–7 AM) of the lights-off period for each 2-week bin for monkeys reared in white light (open bars), red light (red bars), and blue light (blue bars). Error bars represent SEM. ^*^p < 0.05, ^**^p < 0.01, ^***^p < 0.001 for pairwise *post hoc* comparisons between weeks.

Because monkeys demonstrated a significant difference in activity during the first and final hours of the lights-off period, the first hour (7–8 PM), middle 10 h (8 PM–6 AM), and final hour (6–7 AM) of the lights-off period were analyzed separately. Activity data for the first hour, middle 10 h, and final hour of the lights-off period for white, red, and blue light reared monkeys for each 2-week bin from weeks 1–10 and 17–18 are shown in Table 3. Activity during the first hour (7–8 PM) of the lights-off period significantly differed by condition (*p* = 0.004), but not by week (*p* = 0.49; Table 2). Monkeys reared in blue light exhibited significantly lower activity during the first hour of the lights-off period compare to monkeys reared in white light and red light (*p* = 0.01 for both). There was no significant difference in activity between red light and white light reared monkeys (*p* = 0.56; Figure 4A). The interaction effect between condition and week was not significant (*p* = 0.63).

**Table 3:** Average activity (step counts) during the first hour (7–8 PM), middle 10 h (8 PM–6 AM), and final hour (6–7 AM) of the lights-off period for white, red, and blue light reared monkeys by 2-week bins.

Week	Step counts during the first hour (7–8 PM) of the lights-off period			Step counts during the middle 10 h (8 PM–6 AM) of the lights-off period			Step counts during the final hour (6–7 AM) of the lights-off period		
	White light (*n* = 8)	Red light (*n* = 12)	Blue light (*n* = 15)	White light (*n* = 8)	Red light (*n* = 12)	Blue light (*n* = 15)	White light (*n* = 8)	Red light (*n* = 12)	Blue light (*n* = 15)
Weeks 1–2	18 ± 5	13 ± 3	5 ± 1	59 ± 16	56 ± 12	35 ± 7	9 ± 5	7 ± 2	24 ± 5
Weeks 3–4	15 ± 5	9 ± 4	9 ± 4	14 ± 6	41 ± 8	51 ± 20	12 ± 7	13 ± 4	33 ± 7
Weeks 5–6	16 ± 5	16 ± 3	6 ± 2	9 ± 3	31 ± 6	38 ± 10	10 ± 5	15 ± 5	47 ± 8
Weeks 7–8	25 ± 8	19 ± 4	5 ± 2	12 ± 4	40 ± 7	33 ± 9	13 ± 5	16 ± 2	66 ± 9
Weeks 9–10	17 ± 4	15 ± 7	7 ± 2	16 ± 5	34 ± 9	46 ± 17	13 ± 5	19 ± 3	79 ± 11
Weeks 17–18	16 ± 5	23 ± 9	11 ± 3	15 ± 4	13 ± 3	20 ± 7	9 ± 4	21 ± 8	21 ± 5
Average (all weeks)	18 ± 5	16 ± 5	7 ± 3	20 ± 8	35 ± 8	37 ± 13	11 ± 5	15 ± 4	47 ± 9

Data are represented as mean ± SEM.

In contrast, activity during the middle 10 h (8 PM–6 AM) of the lights-off period significantly differed based on week (*p* = 0.001), but not by condition (*p* = 0.22; Table 2). Thus, there were no significant differences in activity during the middle 10 h of the lights-off period between white, red, and blue light reared monkeys. During the middle 10 h of the lights-off period, the greatest activity was observed during weeks 1–2, and the lowest activity was observed during weeks 17–18. Activity during weeks 1–2 was significantly greater than during weeks 5–6, 7–8, and 17–18 (*p* < 0.05 for all; Figure 4B). There were no significant differences in activity during the middle 10 h of the lights-off period between any other 2-week bins (*p* > 0.05 for all). There was no significant interaction effect between condition and week (*p* = 0.38).

Activity during the final hour (6–7 AM) of the lights-off period significantly differed by condition (*p* < 0.001) and week (*p* < 0.001), and there was a significant interaction effect between condition and week (*p* < 0.001; Table 2). Overall, monkeys reared in blue light exhibited significantly greater activity during the final hour of the lights-off period compared to both white light and red light reared monkeys (*p* < 0.001 for both). There was no significant difference in activity between red light and white light reared monkeys (*p* = 0.58; Table 3). *Post hoc* pairwise comparisons on the interaction effect revealed that during weeks 1–2, 5–6, 7–8, and 9–10, monkeys reared in blue light exhibited significantly greater activity during the final hour of the lights-off period compared to monkeys reared in white or red light (*p* < 0.05 for all). During weeks 3–4 and 17–18, activity during the final hour of the lights-off period did not significantly differ between white, red, and blue light reared monkeys (*p* > 0.05 for all). There was no significant difference in activity between white light and red light reared monkeys for any 2-week bin (*p* > 0.05 for all). Blue light reared monkeys exhibited significantly greater activity during weeks 7–8 and 9–10 compared to all other weeks (*p* < 0.01 for all). Greater activity was also observed during weeks 5–6 compared to weeks 1–2, 3–4, and 17–18 (*p* < 0.05 for all). However, white light and red light reared monkeys did not exhibit significant changes in activity during the final hour of the lights-off period between any 2-week bins (*p* > 0.05 for all; Figure 4C).

### Pupil Responses

**Figure 5:**
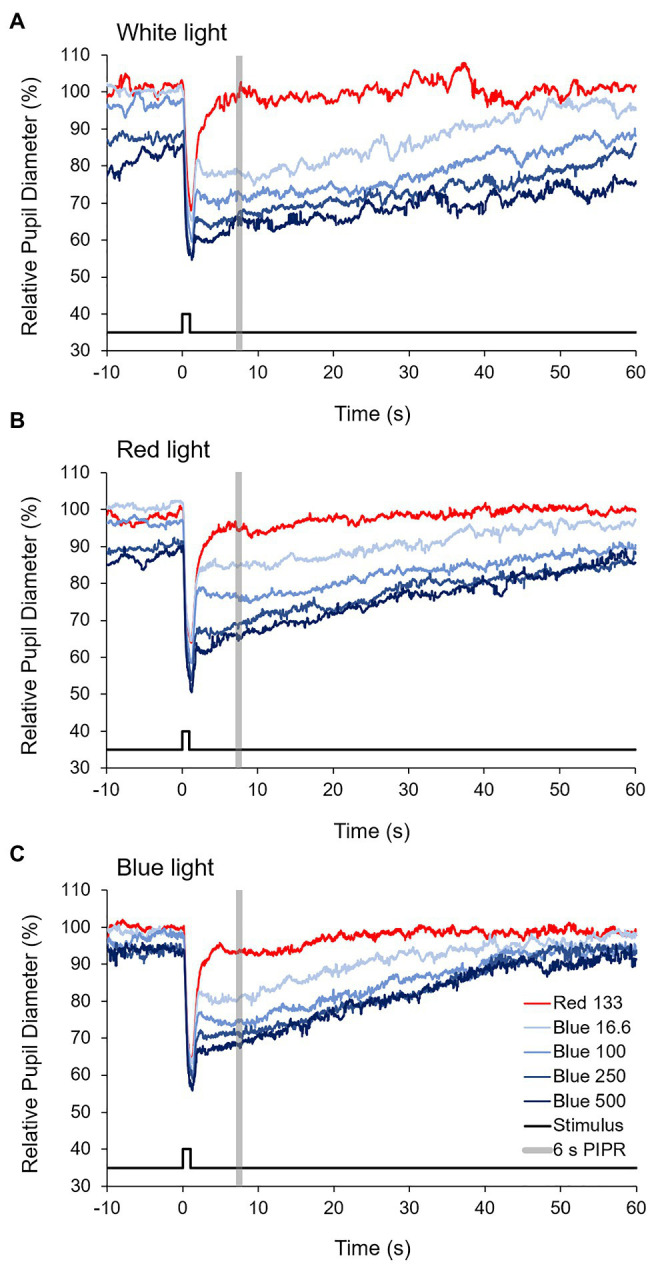
Averaged pupil traces to 1 s red (133 cd/m^2^) and four increasing intensities of blue (16.6, 100, 250, and 500 cd/m^2^) stimuli for **(A)** white light, **(B)** red light, and **(C)** blue light reared monkeys.

**Figure 6:**
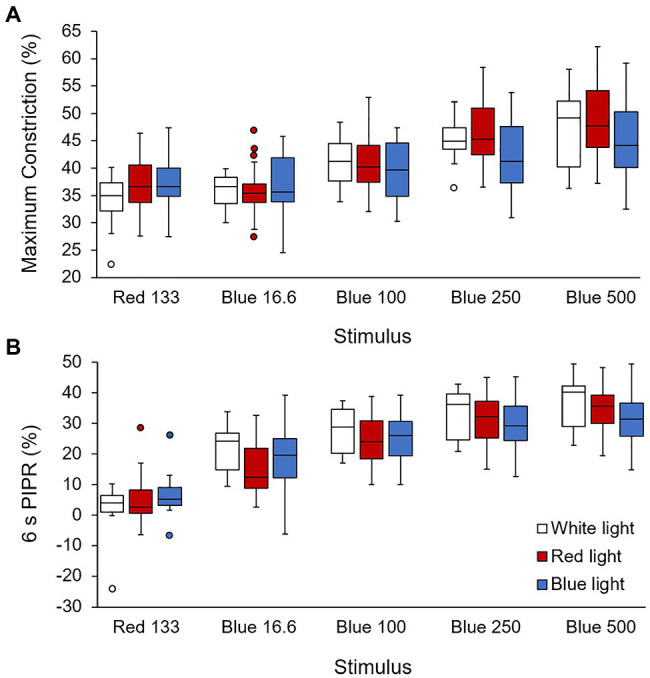
**(A)** Maximum constriction (% change from baseline) and **(B)** the 6 s post-illumination pupil response (PIPR; % change from baseline) for 1 s red (133 cd/m^2^) and four increasing intensities of blue (16.6, 100, 250, and 500 cd/m^2^) stimuli for white light (open bars), red light (red bars), and blue light (blue bars) reared monkeys.

Pupil response data for two monkeys reared in red light were excluded due to excessive fluctuations in pupil diameter. Pupil metrics for the remaining monkeys are shown in Table 4, and averaged pupil traces for white light, red light, and blue light reared monkeys are shown in Figure 5. Baseline pupil diameter, measured during the 4–10 s recording period before the onset of the 1 s red stimulus, for blue light reared monkeys was significantly smaller compared to both white light and red light reared monkeys (*p* = 0.02 and *p* = 0.03, respectively). There was no significant difference in the baseline pupil diameter between white light and red light reared monkeys (*p* = 0.38). The maximum pupil constriction amplitudes to a 1 s red and to all blue stimuli were not significantly different between white, red, and blue light reared monkeys (*p* > 0.05 for all; Figure 6A). Similarly, the 6 s PIPRs to a 1 s red stimulus and to all blue stimuli were not significantly different between white, red, and blue light reared monkeys (*p* > 0.05 for all; Figure 6B).

**Table 4:** Baseline pupil diameter (mm), maximum constriction (% change from baseline), and the 6 s PIPR (% change from baseline) to 1 s red and blue stimuli for white, red, and blue light reared monkeys.

Pupil metric	Stimulus (cd/m^2^)	White light (*n* = 8)	Red light (*n* = 18)	Blue light (*n* = 18)	*p*
Baseline pupil diameter		4.67 ± 0.71	4.38 ± 0.60	3.77 ± 0.88	0.01^*^
Maximum constriction	Red 133	33.66 ± 5.87	37.06 ± 5.03	37.61 ± 4.61	0.18
	Blue 16.6	35.77 ± 3.59	36.02 ± 4.95	36.88 ± 5.92	0.84
	Blue 100	40.88 ± 5.02	41.38 ± 5.79	39.24 ± 5.58	0.51
	Blue 250	44.97 ± 4.90	46.68 ± 6.80	42.24 ± 7.14	0.15
	Blue 500	46.95 ± 8.11	49.17 ± 7.65	44.13 ± 7.85	0.20
6 s PIPR	Red 133	1.11 ± 10.77	5.17 ± 8.42	6.52 ± 6.65	0.34
	Blue 16.6	21.85 ± 8.81	15.09 ± 8.77	19.19 ± 10.89	0.22
	Blue 100	27.55 ± 7.89	24.05 ± 9.04	25.51 ± 8.56	0.63
	Blue 250	32.93 ± 8.61	31.00 ± 9.25	29.63 ± 9.00	0.69
	Blue 500	36.35 ± 9.76	34.34 ± 8.45	31.57 ± 8.29	0.42

Data are represented as mean ± SD.

* Significant difference between conditions.

**Figure 7:**
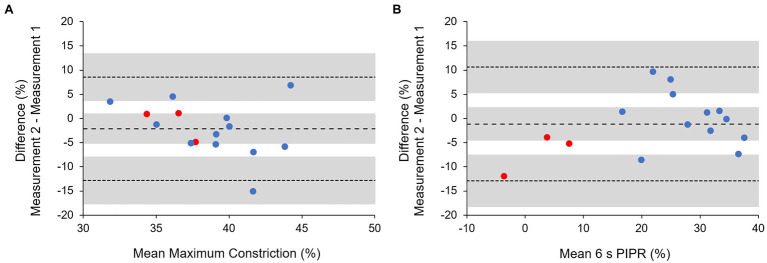
Bland–Altman analysis for **(A)** maximum pupil constriction and **(B)** the 6 s PIPR for a 1 s red stimulus (red symbols) and four intensities of blue stimuli (blue symbols). Dashed and dotted lines represent the mean difference and 95% limits of agreement, respectively. Shaded areas represent the 95% CIs.

For three white light reared monkeys, pupillometry was performed 2–3 months after the first measurement to assess the repeatability. Bland-Altman analysis showed that for maximum constriction, the mean difference between the two measurement sessions was −2.12 ± 5.45% (95% limits of agreement from −12.81 to 8.57%; Figure 7A). For the 6 s PIPR, the mean difference between the two measurement sessions was −1.15 ± 6.01% (95% limits of agreement from −12.93 to 10.63%; Figure 7B). The coefficients of repeatability for maximum constriction and the 6 s PIPR were 11.13 and 11.60%, respectively, indicating that maximum constriction and the 6 s PIPR had similar repeatabilities. The intraclass correlation coefficients (ICCs) for maximum constriction and the 6 s PIPR were 0.23 and 0.89, respectively.

## Discussion

The goal of this study was to examine the impact of long-term exposure to narrowband long- and short-wavelength ambient lighting on behavioral activity patterns and the ipRGC-mediated pupil response in infant monkeys. This study demonstrates that rearing in narrowband short-wavelength light causes greater disruptions in activity rhythms compared to long-wavelength light. Monkeys reared in narrowband red light maintained normal activity patterns, whereas monkeys reared in narrowband blue light exhibited increased nighttime activity. Circadian rhythms are regulated by ipRGCs, which are spectrally tuned to short-wavelength light (Dacey et al., 2005), thus changes in the spectral composition of ambient lighting may influence the sensitivity of the ipRGCs. However, pupil responses measured late in the rearing period, after approximately 8 months, revealed that the PIPR, which is a measure of intrinsic ipRGC activity, was not affected by long-term exposure to narrowband ambient lighting. Normal activity patterns were also observed after approximately 4 months of exposure to narrowband blue light, suggesting that the circadian system can adapt after long-term exposure to narrowband ambient lighting.

The influence of the wavelength of light on diurnal and nocturnal locomotor activity has been previously examined in rodents (van der Merwe et al., 2019). Authors reported that both diurnal and nocturnal rodents raised under a short-wavelength light/dark cycle exhibited lower diurnal activity and greater nocturnal activity compared to rodents raised under a long-wavelength light/dark cycle. In contrast to the results observed in rodents, we found that narrowband red or blue light did not significantly affect daytime activity patterns in infant monkeys. There was a non-significant trend for blue light reared monkeys to exhibit greater daytime activity compared to monkeys reared in red light for all weeks. This trend is opposite to the results reported in rodents (van der Merwe et al., 2019), which may reflect differences in the irradiance levels used, differences in the sensitivity to different wavelengths of light in different species, or differences between nocturnal rodents and diurnal primates. Similar to humans, monkeys have three cone types, short-, middle-, and long-wavelength sensitive cones with maximum spectral sensitivities at approximately 430, 531, and 561 nm, respectively (Baylor et al., 1987). In contrast, rodents have two cone types with maximum spectral sensitivities at approximately 360 and 500 nm (Jacobs et al., 1991; Allen et al., 2020). The lack of a significant difference in daytime activity levels between groups may be due to the high inter-individual variability in activity levels observed in monkeys. Nevertheless, there were consistent age-related effects on daytime activity patterns. During the first 10 weeks of the experimental period, daytime activity levels increased with increasing age, but by weeks 17–18, daytime activity levels had decreased. Importantly, long-term exposure to narrowband ambient lighting did not affect the age-related changes in daytime activity patterns, suggesting that altering the spectral composition of light does not affect normal behavioral developmental patterns in daytime activity in infant monkeys.

Age-related effects were also observed in nighttime activity. Activity during the middle 10 h of the lights-off period was significantly lower by weeks 17–18 compared to the first 2 weeks of the experimental period. The relatively low activity during the middle 10 h of the lights-off period indicates that monkeys were, for the most part, asleep during this time. Previous studies have used video surveillance or actimeters to monitor nighttime activity in rhesus monkeys as a measure of sleep disturbance, with greater nighttime activity or awakenings indicating greater sleep disruption (Golub and Hogrefe, 2016; Stanwicks et al., 2017). Thus, reduced nighttime activity during the middle 10 h of the lights-off period after 4 months suggests that sleep improved with age. While there were no significant differences in activity during the middle 10 h of the lights-off period between groups, monkeys reared in narrowband light displayed higher inter-individual variability in nighttime activity, as indicated by larger SEs, compared to monkeys reared in white light. This suggests that narrowband ambient lighting caused greater sleep disturbances for some monkeys but not for others.

On the other hand, nighttime activity during the first and final hour of the lights-off period was significantly affected by narrowband blue light rearing, but not narrowband red light rearing. The circadian system is most sensitive to short-wavelength light; therefore, while short-wavelength light is important for maintaining a healthy circadian system, it also has greater potential to disrupt circadian rhythms (Bonmati-Carrion et al., 2014; Wahl et al., 2019). Several studies in humans have shown that increased exposure to short-wavelength light in the evening suppresses melatonin secretion (Cajochen et al., 2005; Chellappa et al., 2011; Lee et al., 2018), increases alertness at night (Cajochen et al., 2005; Figueiro et al., 2009), and can affect sleep architecture as measured with electroencephalography (Münch et al., 2006; Chellappa et al., 2013). In contrast, filtering out short wavelengths at night has been reported to increase melatonin levels and improve sleep efficacy, quality, and latency in humans (Burkhart and Phelps, 2009; Ayaki et al., 2016; Ostrin et al., 2017; Nagai et al., 2019). Contrary to what we expected, monkeys reared in blue light exhibited lower activity during the first hour and greater activity during the final hour of the lights-off period compared to white and red light reared monkeys, suggesting shorter sleep latency and earlier wake-up time. Shorter sleep latencies and shorter habitual bedrest duration have been associated with greater sleep debt and sleep deprivation in humans (Klerman and Dijk, 2005; Goel et al., 2009). In addition, we found that for monkeys reared in blue light, activity during the final hour of the lights-off period increased throughout the first 10 weeks of the experimental period while activity during the first hour did not significantly change, suggesting that sleep duration reduced over time. The wavelength of light has been shown to affect sleep induction in mice, such that short-wavelength light delays sleep onset and decreases sleep duration compared to longer wavelengths of light, and melanopsin is involved in this response (Pilorz et al., 2016).

Furthermore, light exposure can phase shift circadian rhythms, and the direction and magnitude of the phase shift are dependent on the timing of light exposure. Phase shifts have been assessed with phase response curves to various exposure durations of white light, which were constructed from studies in humans (Khalsa et al., 2003; Kripke et al., 2007; St Hilaire et al., 2012). In general, light exposure in the morning results in a phase advance, whereas light exposure in the evening or at night results in a phase delay. About 6.5 h of exposure to blue light was shown to produce similar phase shifting responses as 6.7 h of 10,000 lux white light in terms of the direction of the response, however, the irradiance of the blue light was low (Rüger et al., 2013). In contrast, Revell et al. (2012) showed that the phase response curve to 1.5 h of blue light has a larger phase advance region, extending into the late afternoon, suggesting that exposure to blue light in the afternoon can also produce a phase advancing effect. Thus, it is possible that continuous exposure to blue light throughout the entire daytime period can phase advance circadian rhythms. As such, the increased activity in the final hour of the lights-off period, indicative of earlier activity onset, observed in monkeys reared in blue light could have been a result of a phase advance in the activity rhythm.

We speculate that increased melanopsin signaling to the ipRGCs in the evening may have played a role in the increase in nighttime activity observed in monkeys reared in narrowband blue light. To assess ipRGC activity *in vivo*, we measured pupil responses to blue stimuli and used the 6 s PIPR as a measure of intrinsic ipRGC activity. The 6 s PIPR was shown to have the lowest intra- and inter-individual variability in humans (Adhikari et al., 2015). We hypothesized that long-term exposure to narrowband red and blue light would significantly alter the sensitivity of the ipRGCs. However, we found that the PIPR did not differ between monkeys reared in white, red, or blue light. There was also no difference in maximum pupil constriction, which is primarily attributed to rod and cone activity (Kardon et al., 2009), between monkeys reared in white, red, or blue light. These results are consistent with a previous study that examined the effects of short-term exposure to narrowband blue and red light on the ipRGC-mediated pupil response. It was demonstrated that 1 h of exposure to narrowband blue or red light in the morning does not differentially affect the ipRGC-mediated pupil response in adults (Lou and Ostrin, 2020). Interestingly, it can be observed that pupil redilation back to baseline was faster for monkeys reared in blue light. For monkeys reared in white and red light, the pupil did not fully redilate back to baseline during the 60 s interstimulus interval for the three higher intensity blue stimuli, whereas for monkeys reared in blue light, the pupil redilated back to baseline during the 60 s interstimulus interval following all 1 s blue stimuli. It has been shown in humans that the sustained PIPR can last up to 83 s for a 1 s blue stimulus, with high intra- and inter-individual variation (Adhikari et al., 2015). Whether the faster pupil redilation observed in monkeys reared in blue light was due to altered ipRGC function or other factors, such as differences in baseline pupil diameter, requires further investigation.

Studies in humans have shown that ipRGC activity is influenced by environmental light exposure. Münch et al. (2016, 2017) demonstrated that the ipRGC-driven pupil response differs between winter and summer seasons in adults, presumably due to differences in light exposure given that the intensity, spectral composition, and photoperiod or duration of light exposure all vary with season (Thorne et al., 2009). Moreover, in children, the amplitude of the PIPR was shown to be associated with the amount of light exposure measured over the previous 24 h (Ostrin, 2018). However, the impact of the spectral composition of light on ipRGC activity was not independently assessed given that red and blue light exposure were highly correlated with the amount of white light exposure. It is possible that other factors, such as the intensity or total duration of light exposure, influenced ipRGC activity. Maintaining monkeys on a regular light/dark cycle in a controlled laboratory environment allowed us to specifically examine the impact of the spectral composition of ambient lighting on ipRGC activity, without the influence of photoperiod or intensity.

Although maximum constriction and PIPR amplitudes did not differ, monkeys reared in blue light had smaller baseline pupil diameters compared to monkeys reared in white and red light. It is possible that long-term exposure to blue ambient lighting influenced baseline pupil diameter due to increased melanopsin signaling to the ipRGCs. The smaller baseline pupil diameters may also contribute to the faster pupil redilation observed in monkeys reared in blue light. Further studies are warranted to better understand the effects of narrowband lighting and the role of ipRGCs on long-term pupil diameter regulation. Pupil responses are dependent on the baseline pupil diameter; therefore, we compared relative pupil metric values rather than absolute millimeter values to minimize the effect of individual differences in baseline pupil diameter on pupil metric values (Joyce et al., 2016; Kelbsch et al., 2019). Monkeys reared in red light had similar baseline pupil diameters to monkeys reared in white light. Hung et al. (2018) previously found that the pupil diameters of monkeys reared in red light were larger compared to the pupil diameters of monkeys reared in white light; however, pupil diameter was measured while monkeys were in the red or white lighting, whereas here, we measured baseline pupil diameter in the dark.

Neither activity patterns nor pupil responses were significantly affected in monkeys reared in narrowband red light, suggesting that extrinsic input from rod and cone photoreceptors may compensate for the lack of direct melanopsin input to the ipRGCs. Evidence suggests that rod and cone contributions to the non-image forming responses to light are mediated through the ipRGCs (Güler et al., 2007). Mice lacking the melanopsin gene are still able to entrain to the light/dark cycle (Panda et al., 2002; Ruby et al., 2002), whereas genetic ablation of the ipRGCs results in severe deficits in circadian photoentrainment (Güler et al., 2008; Hatori et al., 2008). Our results provide further evidence that normal circadian activity can be maintained without direct melanopsin activation from short-wavelength light. Furthermore, results from *in vitro* studies show that ipRGCs can adjust their sensitivity to background light levels over time, providing evidence that ipRGCs are capable of light adaptation (Wong et al., 2005; Do and Yau, 2013). Studies involving long-term alteration of ambient lighting have demonstrated that the circadian system can adapt to the spectral composition of light. Giménez et al. (2014) reported that using soft orange contact lenses to reduce short-wavelength light by approximately 50% for 2 weeks does not affect the melatonin rhythm or sleep parameters in humans. Similarly, Domagalik et al. (2020) found no changes in evening melatonin levels and sleep parameters after filtering out approximately 90% of short-wavelength light for 4 weeks; however, performance on cognitive tasks was significantly decreased. In our study, we found that normal nighttime activity patterns resumed after approximately 4 months of narrowband ambient light exposure. Normal activity patterns measured later in the rearing period suggest that the circadian system adapted to the narrowband light conditions, possibly mediated by an adjustment of sensitivity of the ipRGCs. Further studies are required to determine whether adaptation of the ipRGCs to environmental light levels are responsible for mediating adaptation of behavioral and physiological functions to different ambient light conditions.

In addition to comparing the pupil responses between monkeys reared in white, red, and blue light, we also assessed the repeatability of the pupil metrics in three monkeys reared in white light, with approximately 2–3 months between measurement sessions. Repeatability of the PIPR has been previously assessed in humans, but not in monkeys. van der Meijden et al. (2015) determined the test-rest reliability of the PIPR measured on 2 consecutive days and reported an ICC of 0.87. Bruijel et al. (2016) assessed the repeatability of the PIPR between summer and winter measurements and reported an ICC of 0.80. Both of these studies used a protocol with a 5 min light stimulus and found good test-retest reliability for the PIPR. Using a 1 s light stimulus protocol, Flanagan et al. (2020) determined the repeatability of maximum constriction and the 6 s PIPR in humans and reported an ICC of 0.77 and 0.63 for maximum constriction and the 6 s PIPR to a blue stimulus, respectively. We also found high test-retest reliability for the 6 s PIPR in monkeys (ICC = 0.89). However, the test-retest reliability for maximum constriction was poor (ICC = 0.23). The low ICC may be due to the lower variability between monkeys for maximum constriction values (Bland and Altman, 1990). Given that the PIPR to a blue stimulus is attributed to intrinsic melanopsin activity (Gamlin et al., 2007); the higher variability in PIPR measurements may reflect greater individual differences in the sensitivity of the melanopsin system. In contrast, maximum constriction and the 6 s PIPR had similar agreements, determined with Bland–Altman analysis, with a coefficient of repeatability between 11 and 12% for both pupil metrics. This is consistent with the agreements reported in previous studies conducted in humans (van der Meijden et al., 2015; Bruijel et al., 2016; Flanagan et al., 2020). Therefore, while there was higher inter-individual variability in PIPR measurements, both maximum constriction and the PIPR can be measured with similar repeatabilities.

A limitation of this study is that Fitbits were utilized to track activity in monkeys. Fitbits are wrist worn devices to measure step counts in humans. Fitbits record activity as “step counts” and are not specifically designed for use on monkeys. Thus, the step counts should be considered relative activity levels. Using more precise measurement techniques, such as with a research grade actigraphy device or implantable telemetry, would allow for a more accurate measurement of activity patterns as well as sleep parameters. Additionally, measurement of other circadian markers, such as melatonin and cortisol levels, would be helpful in understanding the influence of experimental light exposures on potential circadian disruption. Another limitation of this study is that we did not measure pupil responses at the start of the experimental period, but only after approximately 8 months. Thus, we can only speculate that the ipRGCs had adjusted their sensitivity to adapt to the spectral composition of light. Moreover, pupil responses were always measured in the morning. Given that only nighttime activity patterns were disrupted, it is possible that differences in ipRGC activity might have occurred in the evening. Future long-term studies should consider measuring ipRGC activity throughout the experimental period and at multiple times of day to better understand whether the ipRGCs can adjust their sensitivity under different light exposure conditions or to measure potential age-related changes in ipRGC activity.

In summary, our results show that altering the wavelength of ambient lighting impacts nighttime behavioral patterns, but does not significantly alter daytime behavioral patterns. Narrowband short-wavelength light causes greater circadian disruption than narrowband long-wavelength light through the disruption of nighttime activity patterns. However, the circadian system adapts after long-term exposure to narrowband ambient lighting. The ipRGCs may be involved in this adaptation mechanism, allowing behavioral and physiological responses to light to function normally under a variety of ambient light conditions.

## Data Availability Statement

The raw data supporting the conclusions of this article will be made available by the authors, without undue reservation.

## Ethics Statement

The animal study was reviewed and approved by the Institutional Animal Care and Use Committee at the University of Houston.

## Author Contributions

LL: data analysis and drafting the manuscript. BA: initial data analysis. BA, L-FH, ZS, and KB: Fitbit data collection and animal care. LL, LO, and KB: pupil experiments. All authors contributed to the article and approved the submitted version.

## Conflict of Interest

LL, BA, L-FH, ZS, KB, and LO declare that the research was conducted in the absence of any commercial or financial relationships that could be construed as a potential conflict of interest. ES holds patents for optical treatment strategies for myopia and is a consultant for SightGlass Vision Inc., Treehouse Eyes Inc., and Vision CRC USA on issues related to myopia management.

## Publisher’s Note

All claims expressed in this article are solely those of the authors and do not necessarily represent those of their affiliated organizations, or those of the publisher, the editors and the reviewers. Any product that may be evaluated in this article, or claim that may be made by its manufacturer, is not guaranteed or endorsed by the publisher.

## References

[ref1] Adhikari P. Zele A. J. Feigl B. (2015). The post-illumination pupil response (PIPR). Invest. Ophthalmol. Vis. Sci. 56, 3838–3849. doi: 10.1167/iovs.14-16233, PMID: 26066752

[ref2] Allen A. E. Mouland J. W. Rodgers J. Baño-Otálora B. Douglas R. H. Jeffery G.. (2020). Spectral sensitivity of cone vision in the diurnal murid *Rhabdomys pumilio*. J. Exp. Biol. 223:jeb215368. doi: 10.1242/jeb.215368, PMID: 32371443 PMC7272338

[ref3] Arumugam B. Hung L.-F. Ostrin L. A. She Z. Smith E. L. (2018). Effects of long-wavelength lighting on activity patterns and the pupil in infant rhesus monkeys. Invest. Ophthalmol. Vis. Sci. 59:5043. 10.1167/iovs.15-17025 PMC4604957 26447984

[ref4] Ayaki M. Hattori A. Maruyama Y. Nakano M. Yoshimura M. Kitazawa M.. (2016). Protective effect of blue-light shield eyewear for adults against light pollution from self-luminous devices used at night. Chronobiol. Int. 33, 134–139. doi: 10.3109/07420528.2015.1119158, PMID: 26730983

[ref5] Baylor D. A. Nunn B. J. Schnapf J. L. (1987). Spectral sensitivity of cones of the monkey *Macaca fascicularis*. J. Physiol. 390, 145–160. doi: 10.1113/jphysiol.1987.sp016691, PMID: 3443931 PMC1192171

[ref6] Berson D. M. Dunn F. A. Takao M. (2002). Phototransduction by retinal ganglion cells that set the circadian clock. Science 295, 1070–1073. doi: 10.1126/science.1067262, PMID: 11834835

[ref7] Bland J. M. Altman D. G. (1986). Statistical methods for assessing agreement between two methods of clinical measurement. Lancet 327, 307–310. doi: 10.1016/S0140-6736(86)90837-8 2868172

[ref8] Bland J. M. Altman D. G. (1990). A note on the use of the intraclass correlation coefficient in the evaluation of agreement between two methods of measurement. Comput. Biol. Med. 20, 337–340. doi: 10.1016/0010-4825(90)90013-F 2257734

[ref9] Bonmati-Carrion M. A. Arguelles-Prieto R. Martinez-Madrid M. J. Reiter R. Hardeland R. Rol M. A.. (2014). Protecting the melatonin rhythm through circadian healthy light exposure. Int. J. Mol. Sci. 15, 23448–23500. doi: 10.3390/ijms151223448, PMID: 25526564 PMC4284776

[ref10] Brainard G. C. Hanifin J. P. Greeson J. M. Byrne B. Glickman G. Gerner E.. (2001). Action spectrum for melatonin regulation in humans: evidence for a novel circadian photoreceptor. J. Neurosci. 21, 6405–6412. doi: 10.1523/JNEUROSCI.21-16-06405.2001, PMID: 11487664 PMC6763155

[ref11] Bruijel J. van der Meijden W. P. Bijlenga D. Dorani F. Coppens J. E. Te Lindert B. H. W.. (2016). Individual differences in the post-illumination pupil response to blue light: assessment without mydriatics. Biology 5:34. doi: 10.3390/biology5030034, PMID: 27618116 PMC5037353

[ref12] Burkhart K. Phelps J. R. (2009). Amber lenses to block blue light and improve sleep: a randomized trial. Chronobiol. Int. 26, 1602–1612. doi: 10.3109/07420520903523719, PMID: 20030543

[ref13] Cajochen C. (2007). Alerting effects of light. Sleep Med. Rev. 11, 453–464. doi: 10.1016/j.smrv.2007.07.009, PMID: 17936041

[ref14] Cajochen C. Münch M. Kobialka S. Kräuchi K. Steiner R. Oelhafen P.. (2005). High sensitivity of human melatonin, alertness, thermoregulation, and heart rate to short wavelength light. J. Clin. Endocrinol. Metab. 90, 1311–1316. doi: 10.1210/jc.2004-0957, PMID: 15585546

[ref15] Chellappa S. L. Steiner R. Blattner P. Oelhafen P. Götz T. Cajochen C. (2011). Non-visual effects of light on melatonin, alertness and cognitive performance: can blue-enriched light keep us alert? PLoS One 6:e16429. doi: 10.1371/journal.pone.0016429, PMID: 21298068 PMC3027693

[ref16] Chellappa S. L. Steiner R. Oelhafen P. Lang D. Götz T. Krebs J.. (2013). Acute exposure to evening blue-enriched light impacts on human sleep. J. Sleep Res. 22, 573–580. doi: 10.1111/jsr.12050, PMID: 23509952

[ref17] Dacey D. M. Liao H.-W. Peterson B. B. Robinson F. R. Smith V. C. Pokorny J.. (2005). Melanopsin-expressing ganglion cells in primate retina signal colour and irradiance and project to the LGN. Nature 433, 749–754. doi: 10.1038/nature03387, PMID: 15716953

[ref18] Daley J. T. Turner R. S. Freeman A. Bliwise D. L. Rye D. B. (2006). Prolonged assessment of sleep and daytime sleepiness in unrestrained *Macaca mulatta*. Sleep 29, 221–231. doi: 10.1093/sleep/29.2.221, PMID: 16494090

[ref19] Do M. T. H. Yau K.-W. (2013). Adaptation to steady light by intrinsically photosensitive retinal ganglion cells. Proc. Natl. Acad. Sci. U. S. A. 110, 7470–7475. doi: 10.1073/pnas.1304039110, PMID: 23589882 PMC3645585

[ref20] Domagalik A. Oginska H. Beldzik E. Fafrowicz M. Pokrywka M. Chaniecki P.. (2020). Long-term reduction of short-wavelength light affects sustained attention and visuospatial working memory with no evidence for a change in circadian rhythmicity. Front. Neurosci. 14:654. doi: 10.3389/fnins.2020.00654, PMID: 32719581 PMC7348134

[ref21] Duffy J. F. Czeisler C. A. (2009). Effect of light on human circadian physiology. Sleep Med. Clin. 4, 165–177. doi: 10.1016/j.jsmc.2009.01.004, PMID: 20161220 PMC2717723

[ref22] Emens J. S. Burgess H. J. (2015). Effect of light and melatonin and other melatonin receptor agonists on human circadian physiology. Sleep Med. Clin. 10, 435–453. doi: 10.1016/j.jsmc.2015.08.001, PMID: 26568121 PMC4648706

[ref23] Figueiro M. G. Bierman A. Plitnick B. Rea M. S. (2009). Preliminary evidence that both blue and red light can induce alertness at night. BMC Neurosci. 10:105. doi: 10.1186/1471-2202-10-105, PMID: 19712442 PMC2744917

[ref24] Figueiro M. G. Bullough J. D. Bierman A. Fay C. R. Rea M. S. (2007). On light as an alerting stimulus at night. Acta Neurobiol. Exp. 67, 171–178. PMID: 17691225 10.55782/ane-2007-1645

[ref25] Flanagan S. C. Saunders K. J. Queener H. M. Richardson P. Ostrin L. A. (2020). Effects of mydriatics on rod/cone- and melanopsin-driven pupil responses. Optom. Vis. Sci. 97, 198–206. doi: 10.1097/OPX.0000000000001486, PMID: 32168243 PMC7080325

[ref26] Gamlin P. D. R. McDougal D. H. Pokorny J. Smith V. C. Yau K.-W. Dacey D. M. (2007). Human and macaque pupil responses driven by melanopsin-containing retinal ganglion cells. Vis. Res. 47, 946–954. doi: 10.1016/j.visres.2006.12.015, PMID: 17320141 PMC1945238

[ref27] Giménez M. C. Beersma D. G. Bollen P. van der Linden M. L. Gordijn M. C. (2014). Effects of a chronic reduction of short-wavelength light input on melatonin and sleep patterns in humans: evidence for adaptation. Chronobiol. Int. 31, 690–697. doi: 10.3109/07420528.2014.893242, PMID: 24597610

[ref28] Goel N. Rao H. Durmer J. S. Dinges D. F.﻿ (2009). Neurocognitive consequences of sleep deprivation. Semin. Neurol. 29, 320–339. doi: 10.1055/s-0029-1237117, PMID: 19742409 PMC3564638

[ref29] Golub M. S. Hogrefe C. E. (2016). Sleep disturbance as detected by actigraphy in pre-pubertal juvenile monkeys receiving therapeutic doses of fluoxetine. Neurotoxicol. Teratol. 55, 1–7. doi: 10.1016/j.ntt.2016.02.006, PMID: 26956991 PMC4884518

[ref30] Güler A. D. Altimus C. M. Ecker J. L. Hattar S. (2007). Multiple photoreceptors contribute to nonimage-forming visual functions predominantly through melanopsin-containing retinal ganglion cells. Cold Spring Harb. Symp. Quant. Biol. 72, 509–515. doi: 10.1101/sqb.2007.72.074, PMID: 18522518

[ref31] Güler A. D. Ecker J. L. Lall G. S. Haq S. Altimus C. M. Liao H.-W.. (2008). Melanopsin cells are the principal conduits for rod-cone input to non-image-forming vision. Nature 453, 102–105. doi: 10.1038/nature06829, PMID: 18432195 PMC2871301

[ref32] Hannibal J. Kankipati L. Strang C. E. Peterson B. B. Dacey D. Gamlin P. D. (2014). Central projections of intrinsically photosensitive retinal ganglion cells in the macaque monkey. J. Comp. Neurol. 522, 2231–2248. doi: 10.1002/cne.23588, PMID: 24752373 PMC3996456

[ref33] Hatori M. Le H. Vollmers C. Keding S. R. Tanaka N. Buch T.. (2008). Inducible ablation of melanopsin-expressing retinal ganglion cells reveals their central role in non-image forming visual responses. PLoS One 3:e2451. doi: 10.1371/journal.pone.0002451, PMID: 18545654 PMC2396502

[ref34] Hattar S. Kumar M. Park A. Tong P. Tung J. Yau K.-W.. (2006). Central projections of melanopsin-expressing retinal ganglion cells in the mouse. J. Comp. Neurol. 497, 326–349. doi: 10.1002/cne.20970, PMID: 16736474 PMC2885916

[ref35] Hattar S. Liao H. W. Takao M. Berson D. M. Yau K. W. (2002). Melanopsin-containing retinal ganglion cells: architecture, projections, and intrinsic photosensitivity. Science 295, 1065–1070. doi: 10.1126/science.1069609, PMID: 11834834 PMC2885915

[ref36] Hsieh K. C. Robinson E. L. Fuller C. A. (2008). Sleep architecture in unrestrained rhesus monkeys (*Macaca mulatta*) synchronized to 24-hour light-dark cycles. Sleep 31, 1239–1250. doi: 10.5665/sleep/31.9.1239, PMID: 18788649 PMC2542979

[ref37] Hung L.-F. Arumugam B. She Z. Ostrin L. Smith E. L. 3rd. (2018). Narrow-band, long-wavelength lighting promotes hyperopia and retards vision-induced myopia in infant rhesus monkeys. Exp. Eye Res. 176, 147–160. doi: 10.1016/j.exer.2018.07.004, PMID: 29981345 PMC6215717

[ref38] Hung L.-F. Beach K. She Z. Arumugam B. Ostrin L. Smith E. L. (2020). Effect of narrowband, short-wavelength ambient lighting on refractive development in infant rhesus monkeys. Invest. Ophthalmol. Vis. Sci. 61:7. 10.1167/iovs.15-17025 PMC4604957 26447984

[ref39] Jacobs G. H. Neitz J. Deegan J. F. (1991). Retinal receptors in rodents maximally sensitive to ultraviolet light. Nature 353, 655–656. doi: 10.1038/353655a0, PMID: 1922382

[ref40] Joyce D. S. Feigl B. Zele A. J. (2016). The effects of short-term light adaptation on the human post-illumination pupil response. Invest. Ophthalmol. Vis. Sci. 57, 5672–5680. doi: 10.1167/iovs.16-19934, PMID: 27784072

[ref41] Kardon R. Anderson S. C. Damarjian T. G. Grace E. M. Stone E. Kawasaki A. (2009). Chromatic pupil responses: preferential activation of the melanopsin-mediated versus outer photoreceptor-mediated pupil light reflex. Ophthalmology 116, 1564–1573. doi: 10.1016/j.ophtha.2009.02.007, PMID: 19501408

[ref42] Kelbsch C. Strasser T. Chen Y. Feigl B. Gamlin P. D. Kardon R.. (2019). Standards in pupillography. Front. Neurol. 10:129. doi: 10.3389/fneur.2019.00129, PMID: 30853933 PMC6395400

[ref43] Khalsa S. B. S. Jewett M. E. Cajochen C. Czeisler C. A. (2003). A phase response curve to single bright light pulses in human subjects. J. Physiol. 549, 945–952. doi: 10.1113/jphysiol.2003.040477, PMID: 12717008 PMC2342968

[ref44] Klerman E. B. Dijk D.-J. (2005). Interindividual variation in sleep duration and its association with sleep debt in young adults. Sleep 28, 1253–1259. doi: 10.1093/sleep/28.10.1253, PMID: 16295210 PMC1351048

[ref45] Kripke D. F. Elliott J. A. Youngstedt S. D. Rex K. M. (2007). Circadian phase response curves to light in older and young women and men. J. Circadian Rhythms 5:4. doi: 10.1186/1740-3391-5-4, PMID: 17623102 PMC1988787

[ref46] Lee S. I. Matsumori K. Nishimura K. Nishimura Y. Ikeda Y. Eto T.. (2018). Melatonin suppression and sleepiness in children exposed to blue-enriched white led lighting at night. Physiol. Rep. 6:e13942. doi: 10.14814/phy2.13942, PMID: 30556352 PMC6295443

[ref47] Lewy A. J. Wehr T. A. Goodwin F. K. Newsome D. A. Markey S. P. (1980). Light suppresses melatonin secretion in humans. Science 210, 1267–1269. doi: 10.1126/science.7434030, PMID: 7434030

[ref48] Lockley S. W. Brainard G. C. Czeisler C. A. (2003). High sensitivity of the human circadian melatonin rhythm to resetting by short wavelength light. J. Clin. Endocrinol. Metab. 88, 4502–4505. doi: 10.1210/jc.2003-030570, PMID: 12970330

[ref49] Lou L. Arumugam B. Hung L.-F. Beach K. M. She Z. Smith E. L.. (2021). Effects of narrowband light rearing on activity and the pupil in infant rhesus monkeys. Invest. Ophthalmol. Vis. Sci. 62:1338.

[ref50] Lou L. Ostrin L. A. (2020). Effects of narrowband light on choroidal thickness and the pupil. Invest. Ophthalmol. Vis. Sci. 61:40. doi: 10.1167/iovs.61.10.40, PMID: 32832970 PMC7452857

[ref51] Masuda K. Zhdanova I. V. (2010). Intrinsic activity rhythms in *Macaca mulatta*: their entrainment to light and melatonin. J. Biol. Rhythm. 25, 361–371. doi: 10.1177/0748730410379382, PMID: 20876816

[ref52] Moore R. Y. (1996). Neural control of the pineal gland. Behav. Brain Res. 73, 125–130. doi: 10.1016/0166-4328(96)00083-6, PMID: 8788489

[ref53] Münch M. Kobialka S. Steiner R. Oelhafen P. Wirz-Justice A. Cajochen C. (2006). Wavelength-dependent effects of evening light exposure on sleep architecture and sleep EEG power density in men. Am. J. Physiol. Regul. Integr. Comp. Physiol. 290, R1421–R1428. doi: 10.1152/ajpregu.00478.2005, PMID: 16439671

[ref54] Münch M. Kourti P. Brouzas D. Kawasaki A. (2016). Variation in the pupil light reflex between winter and summer seasons. Acta Ophthalmol. 94, e244–e246. doi: 10.1111/aos.12966, PMID: 26843144

[ref55] Münch M. Ladaique M. Roemer S. Hashemi K. Kawasaki A. (2017). Melanopsin-mediated acute light responses measured in winter and in summer: seasonal variations in adults with and without cataracts. Front. Neurol. 8:464. doi: 10.3389/fneur.2017.00464, PMID: 28955293 PMC5601987

[ref56] Münch M. Léon L. Crippa S. V. Kawasaki A. (2012). Circadian and wake-dependent effects on the pupil light reflex in response to narrow-bandwidth light pulses. Invest. Ophthalmol. Vis. Sci. 53, 4546–4555. doi: 10.1167/iovs.12-9494, PMID: 22669721

[ref57] Nagai N. Ayaki M. Yanagawa T. Hattori A. Negishi K. Mori T.. (2019). Suppression of blue light at night ameliorates metabolic abnormalities by controlling circadian rhythms. Invest. Ophthalmol. Vis. Sci. 60, 3786–3793. doi: 10.1167/iovs.19-27195, PMID: 31504080

[ref58] Ostrin L. A. (2018). The ipRGC-driven pupil response with light exposure and refractive error in children. Ophthalmic Physiol. Opt. 38, 503–515. doi: 10.1111/opo.12583, PMID: 30259538 PMC6202139

[ref59] Ostrin L. A. Abbott K. S. Queener H. M. (2017). Attenuation of short wavelengths alters sleep and the ipRGC pupil response. Ophthalmic Physiol. Opt. 37, 440–450. doi: 10.1111/opo.12385, PMID: 28656675 PMC7229994

[ref60] Ostrin L. A. Strang C. E. Chang K. Jnawali A. Hung L. F. Arumugam B.. (2018). Immunotoxin-induced ablation of the intrinsically photosensitive retinal ganglion cells in rhesus monkeys. Front. Neurol. 9:1000. doi: 10.3389/fneur.2018.01000, PMID: 30542318 PMC6277788

[ref61] Panda S. Sato T. K. Castrucci A. M. Rollag M. D. DeGrip W. J. Hogenesch J. B.. (2002). Melanopsin (opn4) requirement for normal light-induced circadian phase shifting. Science 298, 2213–2216. doi: 10.1126/science.1076848, PMID: 12481141

[ref62] Pilorz V. Tam S. K. E. Hughes S. Pothecary C. A. Jagannath A. Hankins M. W.. (2016). Melanopsin regulates both sleep-promoting and arousal-promoting responses to light. PLoS Biol. 14:e1002482. doi: 10.1371/journal.pbio.1002482, PMID: 27276063 PMC4898879

[ref63] Prayag A. S. Münch M. Aeschbach D. Chellappa S. L. Gronfier C. (2019). Light modulation of human clocks, wake, and sleep. Clocks Sleep 1, 193–208. doi: 10.3390/clockssleep1010017, PMID: 32342043 PMC7185269

[ref64] Revell V. L. Molina T. A. Eastman C. I. (2012). Human phase response curve to intermittent blue light using a commercially available device. J. Physiol. 590, 4859–4868. doi: 10.1113/jphysiol.2012.235416, PMID: 22753544 PMC3487041

[ref65] Ruby N. F. Brennan T. J. Xie X. Cao V. Franken P. Heller H. C.. (2002). Role of melanopsin in circadian responses to light. Science 298, 2211–2213. doi: 10.1126/science.1076701, PMID: 12481140

[ref66] Rüger M. St Hilaire M. A. Brainard G. C. Khalsa S.-B. S. Kronauer R. E. Czeisler C. A.. (2013). Human phase response curve to a single 6.5 h pulse of short-wavelength light. J. Physiol. 591, 353–363. doi: 10.1113/jphysiol.2012.239046, PMID: 23090946 PMC3630790

[ref67] Sahin L. Figueiro M. G. (2013). Alerting effects of short-wavelength (blue) and long-wavelength (red) lights in the afternoon. Physiol. Behav. 116–117, 1–7. doi: 10.1016/j.physbeh.2013.03.014, PMID: 23535242

[ref68] Sahin L. Wood B. M. Plitnick B. Figueiro M. G. (2014). Daytime light exposure: effects on biomarkers, measures of alertness, and performance. Behav. Brain Res. 274, 176–185. doi: 10.1016/j.bbr.2014.08.017, PMID: 25131505

[ref69] She Z. Hung L.-F. Arumugam B. Beach K. M. Smith E. L. (2020). Effects of low intensity ambient lighting on refractive development in infant rhesus monkeys (*Macaca mulatta*). Vis. Res. 176, 48–59. doi: 10.1016/j.visres.2020.07.004, PMID: 32777589 PMC7487012

[ref70] Smith E. L. 3rd Hung L.-F. (1999). The role of optical defocus in regulating refractive development in infant monkeys. Vis. Res. 39, 1415–1435. doi: 10.1016/S0042-6989(98)00229-6, PMID: 10343811

[ref72] Stanwicks L. L. Hamel A. F. Novak M. A. (2017). Rhesus macaques (*Macaca mulatta*) displaying self-injurious behavior show more sleep disruption than controls. Appl. Anim. Behav. Sci. 197, 62–67. doi: 10.1016/j.applanim.2017.09.002, PMID: 29276322 PMC5739341

[ref71] St Hilaire M. A. Gooley J. J. Khalsa S. B. S. Kronauer R. E. Czeisler C. A. Lockley S. W. (2012). Human phase response curve to a 1 h pulse of bright white light. J. Physiol. 590, 3035–3045. doi: 10.1113/jphysiol.2012.227892, PMID: 22547633 PMC3406389

[ref73] Thapan K. Arendt J. Skene D. J. (2001). An action spectrum for melatonin suppression: evidence for a novel non-rod, non-cone photoreceptor system in humans. J. Physiol. 535, 261–267. doi: 10.1111/j.1469-7793.2001.t01-1-00261.x, PMID: 11507175 PMC2278766

[ref74] Thorne H. C. Jones K. H. Peters S. P. Archer S. N. Dijk D.-J. (2009). Daily and seasonal variation in the spectral composition of light exposure in humans. Chronobiol. Int. 26, 854–866. doi: 10.1080/07420520903044315, PMID: 19637047

[ref75] van der Meijden W. P. te Lindert B. H. Bijlenga D. Coppens J. E. Gómez-Herrero G. Bruijel J.. (2015). Post-illumination pupil response after blue light: reliability of optimized melanopsin-based phototransduction assessment. Exp. Eye Res. 139, 73–80. doi: 10.1016/j.exer.2015.07.010, PMID: 26209783

[ref76] van der Merwe I. Bennett N. C. Haim A. Oosthuizen M. K. (2019). Effects of the colour of photophase light on locomotor activity in a nocturnal and a diurnal South African rodent. Biol. Lett. 15:20190597. doi: 10.1098/rsbl.2019.0597, PMID: 31573427 PMC6832176

[ref77] Wahl S. Engelhardt M. Schaupp P. Lappe C. Ivanov I. V. (2019). The inner clock-blue light sets the human rhythm. J. Biophotonics 12:e201900102. doi: 10.1002/jbio.201900102, PMID: 31433569 PMC7065627

[ref78] Weed J. L. Lane M. A. Roth G. S. Speer D. L. Ingram D. K. (1997). Activity measures in rhesus monkeys on long-term calorie restriction. Physiol. Behav. 62, 97–103. doi: 10.1016/S0031-9384(97)00147-9, PMID: 9226348

[ref79] Wong K. Y. Dunn F. A. Berson D. M. (2005). Photoreceptor adaptation in intrinsically photosensitive retinal ganglion cells. Neuron 48, 1001–1010. doi: 10.1016/j.neuron.2005.11.016, PMID: 16364903

[ref80] Zele A. J. Feigl B. Smith S. S. Markwell E. L. (2011). The circadian response of intrinsically photosensitive retinal ganglion cells. PLoS One 6:e17860. doi: 10.1371/journal.pone.0017860, PMID: 21423755 PMC3056772

