# Corrigendum: Long-term narrowband lighting influences activity but not intrinsically photosensitive retinal ganglion cell-driven pupil responses

**DOI:** 10.3389/fphys.2022.1129223

**Published:** 2023-01-10

**Authors:** Linjiang Lou, Baskar Arumugam, Li-Fang Hung, Zhihui She, Krista M. Beach, Earl L. Smith, Lisa A. Ostrin

**Affiliations:** ^1^ College of Optometry, University of Houston, Houston, TX, United States; ^2^ Brien Holden Vision Institute, Sydney, NSW, Australia

**Keywords:** circadian rhythms, intrinsically photosensitive retinal ganglion cells, activity patterns, pupil, light exposure, rhesus monkey

In the published article, there was an error in the **Conflict of Interest** statement.

This sentence previously stated:

“The authors declare that the research was conducted in the absence of any commercial or financial relationships that could be construed as a potential conflict of interest.”

The corrected sentence appears below:

“The authors LL, BA, L-FH, ZS, KB, and LO declare that the research was conducted in the absence of any commercial or financial relationships that could be construed as a potential conflict of interest. ES holds patents for optical treatment strategies for myopia and is a consultant for SightGlass Vision Inc., Treehouse Eyes Inc., and Vision CRC USA on issues related to myopia management.”

The authors apologize for this error and state that this does not change the scientific conclusions of the article in any way. The original article has been updated.

